# Effect of Intersection Angle of Input Channels in Droplet Generators

**DOI:** 10.3390/molecules27061791

**Published:** 2022-03-09

**Authors:** Gi-Beum Kim, Young-Ran Park, Seong-Jong Kim, Kwang-Hyun Park

**Affiliations:** 1 Eouidang Agricultural Company, Wanju-gun 55360, Korea; 2 School of Chemical Engineering, College of Engineering, Jeonbuk National University, Jeonju 54896, Korea; yrpark@gmail.com (Y.-R.P.); ksjong@jbnu.ac.kr (S.-J.K.); 3 BioMedical Science Graduate Program (BMSGP), Chonnam National University, Hwasun 58128, Korea

**Keywords:** droplet generation, intersection angle, flow-focusing (FF) geometries, numerical simulation, droplet diameter

## Abstract

In this paper, we studied the effects of the intersection angle between the inlet channels on the droplet diameter using a COMSOL Multiphysics^®^ simulation. We employed the level-set method to study the droplet generation process inside a microfluidic flow device. A flow-focusing geometry was integrated into a microfluidics device and used to study droplet formation in liquid–liquid systems. Droplets formed by this flow-focusing technique are typically smaller than the upstream capillary tube and vary in size with the flow rates. Different intersection angles were modeled with a fixed width of continuous and dispersed channels, orifices, and expansion channels. Numerical simulations were performed using the incompressible Navier–Stokes equations for single-phase flow in various flow-focusing geometries. As a result of modeling, when the dispersed flow rate and the continuous flow rate were increased, the flow of the continuous flow fluid interfered with the flow of the dispersed flow fluid, which resulted in a decrease in the droplet diameter. Variations in the droplet diameter can be used to change the intersection angle and fluid flow rate. In addition, it was predicted that the smallest diameter droplet would be generated when the intersection angle was 90°.

## 1. Introduction

In recent years, droplet generation in microfluidic channels has become a useful technical platform for diverse applications in the areas of biology, biomedical studies, chemical synthesis, and drug discovery [1,2]. Microscale droplets of liquid can be produced with high throughput and great uniformity when two immiscible fluids are introduced into droplet generation devices, which can be classified into several types: co-flow (coaxial), cross-flow (including T-junctions), and flow-focusing (FF) devices [3]. The droplet formation in these devices is a complicated process resulting from multiple forces such as inertial force, viscous force, and surface tension of two phases (continuous phase and dispersed phase); thus, it depends on many independent parameters such as flow rates, viscosities, and geometric parameters. Herein, ‘continuous or continuous phase flow’ denotes a fluid that allows droplets to flow continuously, while ‘dispersed or dispersed phase flow’ denotes a droplet to be made in a fluid that flows discontinuously. In other words, the distinction between dispersed and continuous is related to the amount of fluid, whereby a large amount becomes continuous and dispersed means a small amount. In traditional studies, the effect of intersection angle on droplet diameter was insignificant; however, in this study, it was expected that intersection angle would have an important effect on droplet diameter. Droplet generation in biochemical processes, as well as in molecular biology processes is very useful, such as in the encapsulation of cells or drugs including cosmetic ingredients. Fluidic barrier production on various microfluidic platforms is important following various chemical bonding networks [4,5]. Therefore, achieving a full understanding of droplet formation is a difficult task that should be based on the consideration of multiple forces and operational/design parameters.

Droplet characterization is typically done by postprocessing images taken with high-speed cameras. Several researchers have carried out experimental studies on the droplet formation process in microfluidic devices. [6,7,8]. Droplet formation mechanisms are classified as squeezing, dripping, and jetting regimes. Garstecki et al. [9], Tice et al. [10], Nie et al. [8], and Graaf et al. [11] suggested that the mechanism changes from “squeezing” to “dripping”. These studies were based on experimental and numerical simulation characterization.

Numerical simulation provides an alternative approach to understanding this complicated phenomenon. Since experimental characterization requires fabrication, operation, and testing of microfluidic droplet generators for diverse design iterations, which are challenging and time-consuming tasks, numerical simulation is advantageous in that it is capable of performing multiple design iterations. In addition, fabrication defects, particularly in the channel section where the droplets are formed, can have a significant impact on the performance of the device, leading to flawed data.

Typically, one of the following methods is employed to model two-phase flow: the volume of fluid (VOF) method, the level-set method, the phase-field method, and the lattice Boltzmann method. The VOF method affects the volume fraction equation and adopts geometric construction to determine the weighted density and viscosity in each computational cell. However, this method can produce large computational errors for microfluidic flow devices with narrow orifices, as it inaccurately captures thin interfaces. Conversely, the level-set method represents the interface using a smooth function, and it is very convenient for calculating the curvature and surface tension forces. It is, therefore, more suitable for modeling the droplet breaking process inside microfluidic flow devices [12]. One of the implicit methods for capturing the interface is the phase-field method, which is also called the diffuse-interface method. The phase-field method is a first-order approximation of the sharp interface model, where a fine grid is required around the interface [13]. In contrast, the level-set approach can be used to exactly locate the interface in a simple fashion. The lattice Boltzmann method (LBM) has shown great potential to model the interfacial interactions, while it incorporates fluid flow as a system feature [14]. It is a pseudo-molecular method tracking evolution of the distribution function of an assembly of molecules and built upon microscopic models and mesoscopic kinetic equations [15]. Its mesoscopic nature can provide many advantages of molecular dynamics [16,17,18], making the LBM especially useful for simulation of droplet dynamics [19,20,21,22].

In this paper, we studied the effects of the intersection angle between the inlet channels on the droplet diameter and frequency using a COMSOL simulation. We employed the level-set method to study the droplet generation process inside a microfluidic flow device. A flow-focusing geometry was integrated into a microfluidics device and used to study droplet formation in liquid–liquid systems. Droplets formed by this flow-focusing technique are typically smaller than the upstream capillary tube and vary in size with the flow rates. The flow-focusing geometry implemented in a microfluidics device involves a liquid that flows into the middle channel and a second immiscible liquid that flows into the two outside channels. The two liquid phases are then forced to flow through a small orifice that is located downstream of the three channels. A phase diagram illustrating the drop size as a function of flow rates and flow rate ratios of the two liquids includes one regime where drop size is comparable to orifice width and a second regime where drop size is dictated by the diameter of a thin “focused” thread, whereby a drop much smaller than the orifice is formed. Both monodisperse and polydisperse emulsions can be produced [23]. Therefore, since no studies have reported on the size of the droplet, we newly investigated the change in droplet diameter predicted using CFD simulation.

## 2. Theory

Laminar flow is the typical flow regimen for microfluidic droplet generators, because the Reynolds number (*Re*), relating the inertia and viscous forces, is scaled so as to be proportional to the length and, therefore, becomes very small in the microscale.

(1) $$Re=\frac{v\rho L}{\mu }$$

where *v* is the velocity (m/s), *μ* is the dynamic viscosity (Pa·s), *ρ* is the density (kg/m^3^), and *L* is the characteristic length (m). The phase field method was selected for use because it can provide continuous tracking of the fluid interface using an energy model that can later be coupled with other physics processes, such as diffusion and heat transfer, for a more complex simulation. The laminar two-phase flow and phase field physics modules were used to model the FF device in two- and three-dimensional spaces [24]. A detailed description of this method can be found in previous research [14].

The study of the droplet generation mechanism is essential in identifying the conditions for stable droplet generation. Capillary number has been used to define dripping and jetting [25].

(2) $$Ca=\frac{\mu v}{\gamma },$$

where *γ* is the surface tension between two immiscible liquids.

Christopher and Anna [3] determined a reasonable estimate of the capillary number *Ca* for FF in planar geometries, as a function of the effective elongation rate *G* = Δ*v/Δz*, where Δ*v* = *v*_orifice_ − *v*_c_ is the velocity difference experienced by the continuous phase liquid as it flows into the orifice, and Δ*z* is the distance from the end of the inner microchannel to the orifice entrance. The capillary number is expressed as follows [23]:

(3) $$Ca=\frac{\mu_{c}Gw_{c}}{\gamma }=\frac{\mu_{c}w_{c}\Delta V}{\gamma \Delta Z}=\frac{\mu_{c}w_{c}Q_{c}}{\gamma h\Delta Z}\left[\frac{1}{w_{or}}-\frac{1}{2w_{c}}\right],$$

where *w_or_* is the width of the orifice.

As previously mentioned, the droplet diameter decreases with an increase in the velocity of the continuous phase, while it increases with an increase in the velocity of the dispersed phase. These results are similar to those from the symmetrically perpendicular rupturing in the FF microfluidic device. By considering the equilibrium between the shear force of the continuous flow and interfacial tension, as well as the influence of the two-phase flow ratio on the shape of the interface, the droplet diameter can be expressed as follows [26,27,28,29].

(4) $$\frac{d_{d}}{w}=k{\left(\frac{Q_{d}}{Q_{c}}\right)}^{\alpha }Ca^{\beta },$$

where *d_d_* is the droplet diameter, *w* is the width of the microchannel, *Q_c_* is the continuous phase flow rate, *Q_d_* is the dispersed phase flow rate, and *Ca* is the capillary number, defined as in Equation (3). The parameters *k*, α, and β are all the same as those determined before using the data in the perpendicular channel.

**Figure 1:**
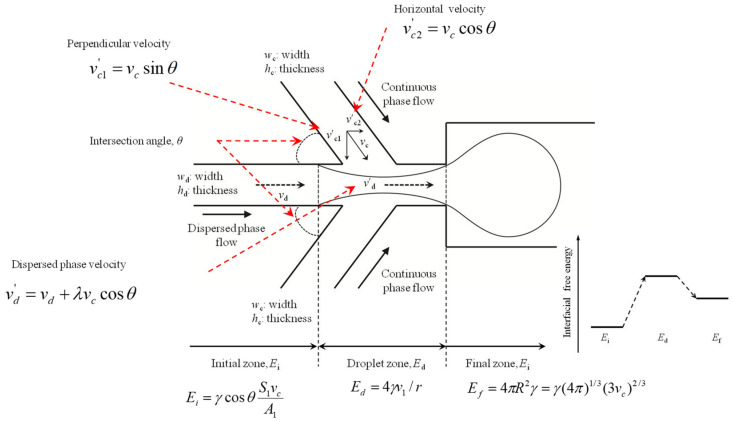
Schematic illustration of the rupture process of droplet generation at an intersection, and a schematic representation of a droplet production device. From the left, two immiscible liquids enter the setup. To the right, an emulsion leaves the device, where the dispersed-phase fluid is dispersed in a continuous-phase fluid. At the bottom, the contents of the interfacial free energy are sketched as an energy-level diagram. The final energy, *E_f_*, is higher than the initial energy, *E_i_*, but there is an even higher intermediate energy, *E_d_*, somewhere within the device.

The influence of the interface angle between dispersed and continuous phase channels on the droplet diameter, *d_d_*, was analyzed as described below. Figure 1 shows a schematic illustration of the rupture process of droplet generation at an intersection between dispersed and continuous phases, which have an interface angle of *θ* [26,27,28]. In the case of a perpendicular FF microfluidic device (*θ* = 90°), the dispersed phase stream is sheared by the continuous phase stream, with a velocity of $v_{d}=Q_{d}/hw_{d},$ until a droplet is formed. The influence that the shape of the microfluidic device exerts on the droplet diameter is proportional, as expressed below [29].

(5) $$d_{d}\propto \frac{v_{d}}{v_{c}}.$$

We considered the influence of the angle between the two entrance channels on this process from two different aspects. First, the perpendicular velocity required to rupture the plug must be modified to $v_{c1}^{\prime }=v_{c}\sin\theta$. However, the influence of the horizontal contribution of the liquid velocity on the dispersed phase flow rate in the formation stage should also be considered. For a channel with intersection angle *θ*, the horizontal part of the continuous phase velocity is $v_{c2}^{\prime }=v_{c}\cos\theta$ [26,27,28]. This velocity is co-current with the dispersed phase flow when *θ* < 90°, but counter-current with the dispersed phase flow when *θ* > 90°. We assumed that the effect of $v_{c2}^{\prime }$ leads to a modification of the dispersed phase velocity [29].

(6) $$v_{d}^{\prime }=v_{d}+\lambda v_{c}\cos\theta ,$$

where *λ* is the efficiency coefficient, which changes as *θ* goes from being less than to greater than 90°. Considering that both horizontal and vertical components of the continuous phase velocity are influenced by the angle of the intersecting channel, *θ*, the droplet diameter can be expressed as follows [26,27,28,29]:

(7) $$\frac{d_{d}}{w}\propto \frac{v_{d}^{\prime }}{v_{c}^{\prime }}=\frac{v_{d}+\lambda v_{c}\cos\theta }{v_{c}\sin\theta }=\frac{Q_{d}}{Q_{c}\sin\theta }+\lambda \cot.$$

Yet another factor affecting droplet diameter is the equilibrium between the shear force of the continuous phase flow, which is characterized by the capillary number [26,27,28]. Due to the influence of the entrance angle on both the shape of the intersection and the equilibrium of the shear force of the continuous phase flow, we assumed that the droplet diameter could be expressed as follows [29]:

(8) $$\frac{d_{d}}{w}=k(\frac{Q_{d}}{Q_{c}\sin\theta }+\lambda \cot\theta {)}^{\alpha }Ca^{\beta },$$

where the parameters *k*, *α*, and *β* are the same as those previously determined using the data from the perpendicular channel.

In Figure 1, two fluids are fed in from the left-hand side, one of which is to be dispersed. To the right, the dispersed phase is present as isolated droplets within the continuous phase. The task of the device is, thus, to decompose the singly connected liquid that is to be dispersed into a set of many unconnected regions of equal volume. It is apparent from the start that the dispersion process is accompanied by an injection of considerable amounts of energy into the system, in order to account for the excess free energy of the interfaces, separating the dispersed from the continuous phase in the emulsion that is finally produced. A schematic sketch of the energy levels shows that *E_i_* corresponds to the initial level, *E_f_* corresponds to the final state, and *E_d_* corresponds to the droplet generation energy (Figure 1). The final state energy can be expressed as follows [30]:

(9) $$E_{f}=4\pi R^{2}\gamma =\gamma {\left(4\pi \right)}^{1/3}{\left(3V_{dr}\right)}^{2/3},$$

where *γ* is the interfacial tension between the two liquids, *V_dr_* is the volume of a droplet, and *R* is radius of the droplet.

In order to calculate the energy of the initial state, *E_i_*, it is necessary to first replace the continuous-phase liquid with the dispersed-phase liquid in the corresponding channel on the left-hand side. The initial energy can then be determined using Young’s equation [31].

(10) $$E_{i}=\gamma \cos\theta \frac{S_{1}V_{dr}}{A_{1}},$$

where *θ* is the contact angle the continuous phase makes at the channel wall in the presence of the dispersed phase. If the channel walls show no preference for one or the other liquid, this becomes *θ* = π/2; thus, *E_i_* = 0. *S*_1_ and *A*_1_ are the surface area and the volume, respectively, per unit length of the feed channel of the dispersed phase liquid. Subsequently, we calculate the interfacial energy of a cylindrical jet of radius *r* [30].

(11) $$E_{d}=2\gamma V_{dr}/r.$$

Dynamical mode selection always ensures that the final droplet volume will be such that *E_f_ < E_d_*.

## 3. Results and Discussion

**Figure 2:**
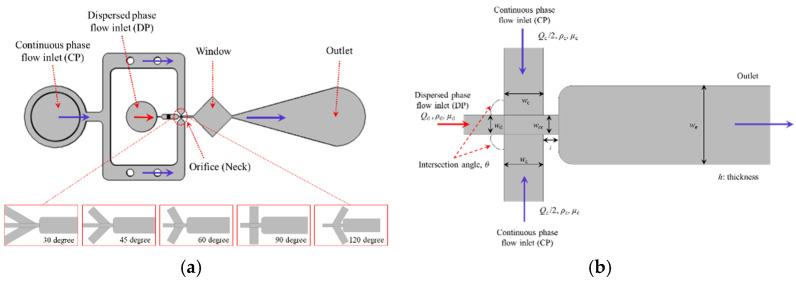
Schematic illustration of the geometric dimensions of the FF droplet generation device for numerical simulation. The device is planar; the channels have a uniform height (typically *h* = 15 μm). The dimensions in the plane of the device are given in the figure (in µm). (**a**) Schematic illustration of droplet generation device. (**b**) Structure of the orifice. In the device shown in the micrograph, the width of the orifice is *w_or_* = 25 μm; a schematic illustration is provided showing the aspect ratio of the height of the device to the widths of the various channels. Typically, the height is much smaller than the lateral dimensions. The oil is delivered via the two outer inlet channels and the water through the channel running along the central line of the device. The stream of water is focused by the streams of oil into the orifice and breaks there to release droplets into the outlet channel. In order to achieve stable operation, the walls of the device have to be preferentially wetted during the continuous phase.

We analyzed the effect of the ratio variation for *v_c_/v_d_* on the diameter of the droplets, the number of droplets, and the representative droplet generation patterns for the systems (Figure 2). In these simulations, we kept the rate of flow of the dispersed phase (water, *Q_d_*) constant. Monitoring how the droplet diameter changed when the flow rate ratios (*v_c_/v_d_*) increased allowed us to determine the dominant mode of break-up. Using the rate-of-flow-controlled break-up generated a prediction that the volume of the droplets is inversely proportional to the ratio of the rates of flow: *V_dr_* ∝ (*Q_c_*/*Q_d_*)^−1^. The shearing mechanism relates the diameter of the droplet to the reciprocal of the capillary number, which yields a much stronger dependence on the ratio of the rates of flow *V_dr_* ∝ (*Q_c_*/*Q_d_*)^−1^. We found that increasing the ratio (*v_c_*/*v_d_*) of the flow rates of the liquids resulted in a decrease in the diameter of the droplets. However, increasing the ratio (*v_c_*/*v_d_*) of the flow rates of the liquids resulted in an increase in the number of droplets. Decreases in the diameters were nonlinear (exponential) in *v_c_*/*v_d_* for all intersection angles. However, increases in the droplet numbers were nonlinear (exponential) in *v_c_/v_d_* for all intersection angles.

FF is the most popular method for generating microdroplets. The geometry of FF is a cross that includes three inlets and one outlet. Two of the inlets are used for carriers, such as oil. The two carrier inlets are usually set at 180° with respect to one another. This takes advantage of the symmetric structure, which provides the same pressure, flow inertia, and intersectional area to both sides. In this way, droplet generation can be well controlled using the intersection angle of the input channel and the flow rate, by taking into consideration its inlet flow inertia and interfacial tension.

As the flow rate of the continuous phase increases and the flow rate of the dispersed phase is kept constant, the diameter of the detaching droplets becomes smaller, as suggested by Equation (3). Therefore, it will eventually reach the diameter of the outlet (dripping regime). Increasing the flow velocity of the continuous phase even further will ensure that the injected phase is no longer able to fulfill the detachment condition.

**Figure 3:**
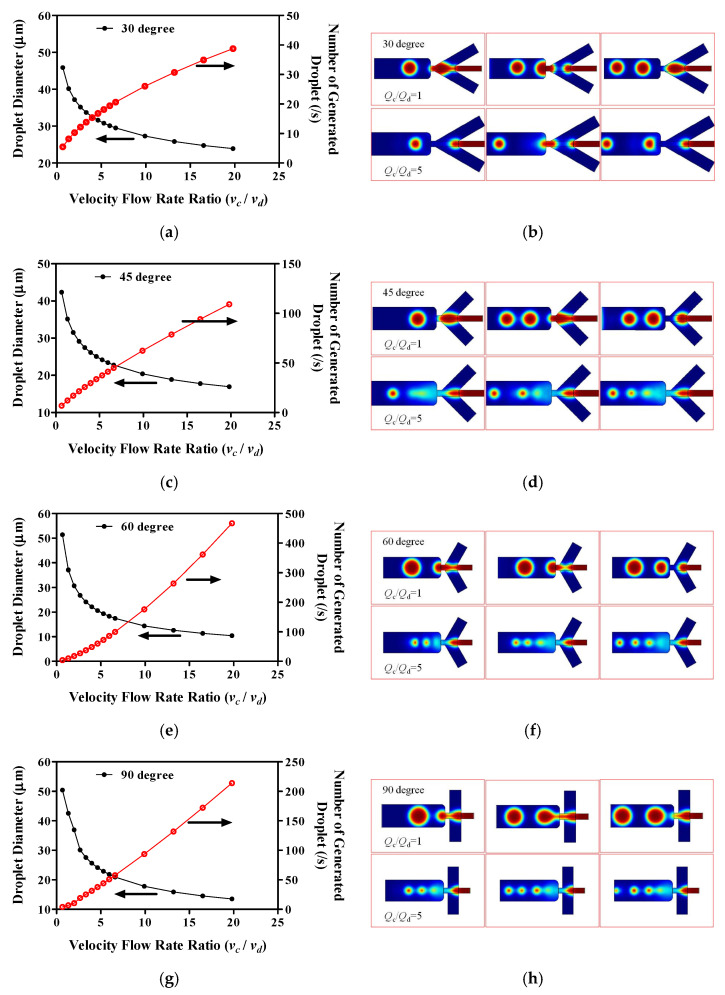

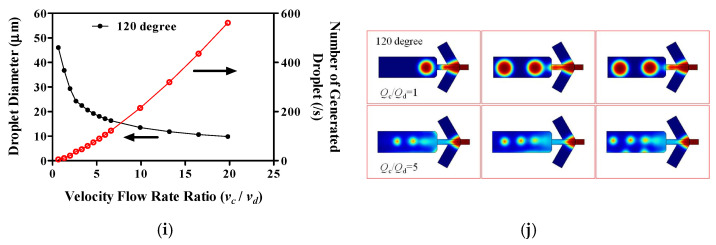
Variation in the droplet diameter and velocity flow rate ratio of the continuous phase flow rate and the dispersed phase flow rate with different intersection angles of the continuous phase channel, plotted as a function of the water-to-oil flow rate ratio, *v_c_/v_d_* (**a**,**c**,**e**,**g**,**i**). Snapshots of droplets formed with different intersection angles of the continuous phase channel under the same hydrodynamic conditions (*v_c_/v_d_* = 1 to 20, *Q_d_* = 1 μL/h) (**b**,**d**,**f**,**h**,**j**). The droplet generation and flow pattern results of simulation modeling for different intersection angles with a fixed width of continuous (*w_c_*) and dispersed (*w_d_*) channels, orifices (*w_o_*), and expansion channels (*w_e_*). (**a**) Intersection angle 30°; (**c**) intersection angle 45°; (**e**) intersection angle 60°; (**g**) intersection angle 90°; (**i**) intersection angle 120°.

We analyzed the flow patterns for our simulation modeling of each type (Figure 3b,d,f,h,j). The droplet formation for the different total flow rates shows a constant value of *Q_c_/Q_d_*, where *Q_c_* is the flow rate of the continuous phase (oil phase) and *Q_d_* is the flow rate of the dispersed phase (water phase); the different models showed different continuous phase flow rates, with a fixed dispersed phase rate of 1 μL/h. We found similar characteristics in all of the device types. When the flow of the dispersed-phase fluid starts, there is a cone-shaped pattern at the intersection of the dispersed-phase fluid and the continuous-phase fluid for all flow rate conditions. The patterns formed from the droplet can be classified into three types: squeezing, dripping, and jetting. In our model, the droplet breaking process is dominated by a squeezing regime, and monodispersed droplets are generated. As the dispersed phase propagates to the entrance of the orifice with a parabolic shape, it blocks the channel of the continuous phase, thus building a high pressure upstream. This high pressure squeezes the dispersed phase into the expanding channel and forms a visible neck inside the orifice. Due to the high pressure and viscous stress exerted by the continuous phase, the neck collapses and a droplet larger than the orifice is separated from the dispersed phase. The surface tension retracts the interface outside of the orifice, and the same cycle is then repeated.

**Figure 4:**
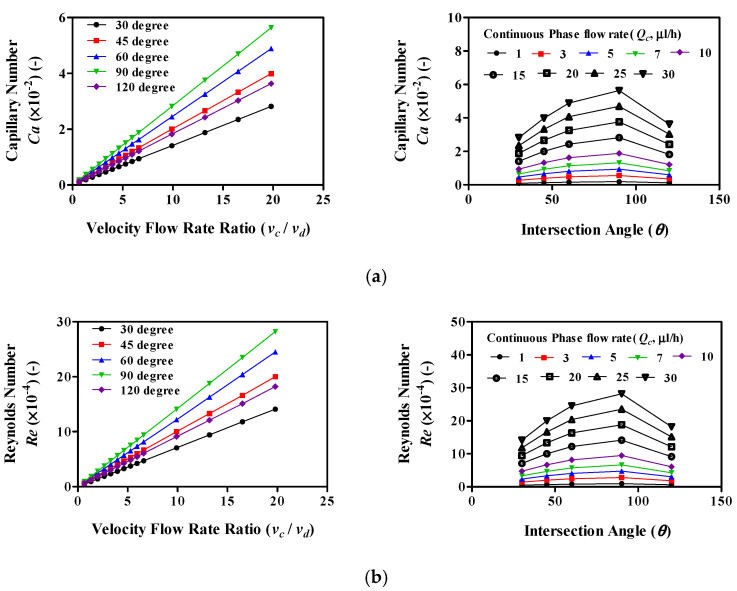
The relationship between the velocity flow rate ratio and the capillary number and Reynolds number for each device. Changes of profiles of the (**a**) Capillary number and (**b**) Reynolds number by increases of velocity flow rate ratio (*v_c_/v_d_*) (***left panel***) and intersection angle (*θ*) (***right panel***).

We subsequently examined the relationship between the velocity flow rate ratio and the capillary number and Reynolds number for each device (Figure 4). The capillary number (*Ca*) and Reynolds number (*Re*) can be described by Equations (2) and (3). The *Ca* and *Re* increase as the velocity flow rate ratio increase. The *Ca* and *Re* increase when the intersection angle is smaller than 90°, but there is a decrease when the intersection angle is greater than 90°. The *Ca* is the same for intersection angles of 60° and 120°, as is the *Re*. This reason is that the *Ca* and *Re* are affected by the flow rate of the continuous phase flow and intersection angle (*θ*) of the continuous phase channel. Therefore, as the flow rate of the continuous phase flow increases, the *Ca* and *Re* are increased. Moreover, when the intersection angle (*θ*) of the continuous phase channel increases, the *Ca* and *Re* are increased. However, even if the intersection angle increases, the *Ca* and *Re* do not always increase. The intersection angles have a decisive effect on the *Ca* and *Re* in a sine wave form. In other words, the *Ca* and *Re* increase when *θ* < 90°; hence, the *Ca* and *Re* decrease when *θ* > 90°. Therefore, when θ is 60° and 120°, they have the same value.

When the capillary number has a low value (*Ca* < 10^−2^), the interfacial force is higher than the shear stresses, and the pressure drop across the forming droplet dominates; therefore, dripping occurs. When the capillary number has a high value (*Ca* > 10^−2^), the shear stress starts to dominate the interfacial force, causing jetting [12]. In our modeling results, the *Ca* was 9 × 10^−4^ to 6.54 × 10^−2^. Therefore, at low values of the *Ca* (<10^−2^), the droplet was generated when dripping occurred; however, when the *Ca* (>10^−2^) value was high, the forming droplet was generated when jetting occurred.

The two most common strategies use FF geometries. In general, the fluid phase that is to be dispersed is brought into a microchannel using a pressure-driven flow, while the flow of the second immiscible carrier liquid is driven independently. These two phases meet at an orifice (or neck, junction), where the local flow field, determined by the geometry of the orifice and the flow rates of the two fluids, deforms the interface. Eventually, droplets pinch off from the dispersed phase finger via free surface instability. The pinch-off of droplets is largely dictated by the competition between the viscous shear stresses, which are acting to deform the liquid interface, and capillary pressure, which is acting to resist the deformation, expressed by Ca. This number ranges between 10^−3^ and 10 for most microfluidic droplet formation devices. Quantitative predictions of the regimes of drop formation and the drop diameter still pose a challenge, although significant progress has been made in both analytical and numerical studies [16,17,18,21].

**Figure 5:**
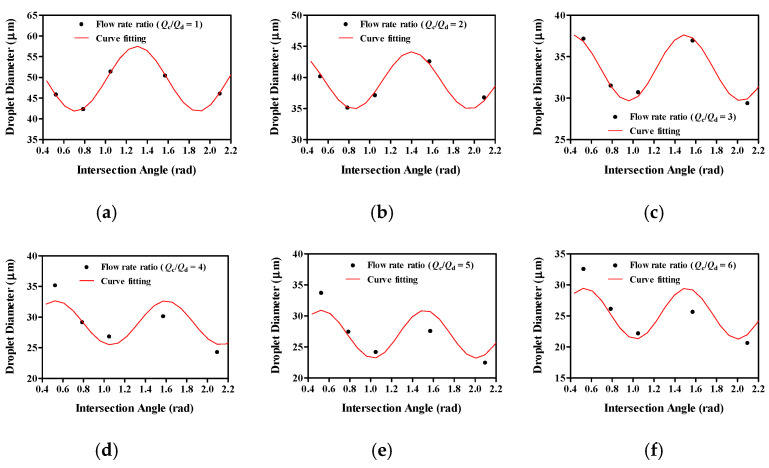
The relationship between intersection angle and droplet diameter for various volume flow rate ratios. The dot is the droplet diameter according to the COMSOL Multiphysics simulation result, and the red wave is the nonlinear curve fitting result of the droplet diameter. Representative droplet diameter changes by increases of intersection angle on various flow rate ratio at (**a**) *Q_c_/Q_d_* = 1, (**b**) *Q_c_/Q_d_* = 2, (**c**) *Q_c_/Q_d_* = 3, (**d**) *Q_c_/Q_d_* = 4, (**e**) *Q_c_/Q_d_* = 5 and (**f**) *Q_c_/Q_d_* = 6.

Figure 5 shows the relationship between the intersection angle and droplet diameter. Our modeling results showed that the droplet diameter decreased with intersection angles of 30° and 45°, increased with intersection angles of 60° and 90°, and decreased with an intersection angle of 120° at a low flow rate ratio (*Q_c_/Q_d_* = 1 and 2). However, as the flow rate ratio exceeded 3, the droplet diameter decreased with intersection angles of 30°, 45°, and 60°, increased with an intersection angle of 90°, and decreased with an intersection angle of 120°. Results of modeling the droplet diameter changes with intersection angle revealed that droplet diameter changed into a sine wave form.

**Figure 6:**
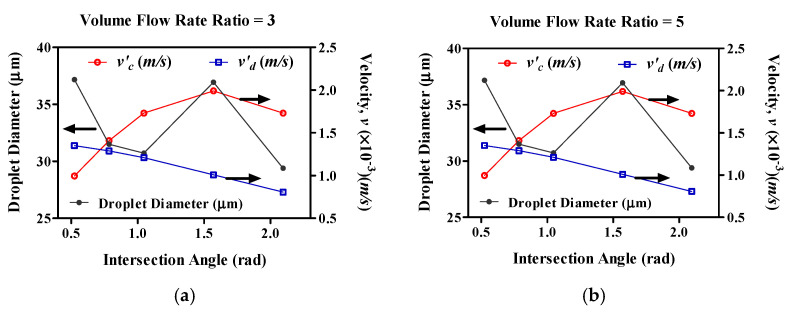
The relationship between intersection angle and droplet diameter, using a modification of the continuous flow rate ($v_{c}^{\prime }$) and dispersed flow rate ($v_{d}^{\prime }$). (**a**) *v_c_/v_d_* = 3; (**b**) *v_c_/v_d_* = 5.

We determined the relationship between the intersection angle and the droplet diameter, using a modification of the continuous flow rate ($v_{c}^{\prime }$) and dispersed flow rate ($v_{d}^{\prime }$) for volume flow rate ratios of 3 and 5 (Figure 6). The $v_{d}^{\prime }$ is the modification of the dispersed phase rate, and the $v_{c}^{\prime }$ is the modification of the continuous phase rate at the droplet generator orifice. As shown Figure 1, dispersed-phase fluid incurred rupture, due to the continuous-phase fluid at this point. Therefore, the dispersed-phase fluid velocity increased because of the continuous-phase fluid. This rate is co-current with the dispersed phase flow when *θ* < 90°; hence, this rate increases when the intersection angle increases. Conversely, increasing this rate has an effect on the rate variations, and the $v_{d}^{\prime }$ decreases when the $v_{c}^{\prime }$ increases at *θ* < 90°. This rate is counter-current with the dispersed phase flow when *θ* > 90°. In addition, this rate decrease has an effect on the droplet diameter and the number of droplets; hence, droplet diameter decreases and the number of droplets increases when this rate ($v_{d}^{\prime }$) decreases This reason is that the continuous flow rate ($v_{c}^{\prime }$) and dispersed flow rate ($v_{d}^{\prime }$) are affected by the flow rate of the continuous phase flow and intersection angle (*θ*) of the continuous phase channel. Therefore, when the flow rate of continuous phase flow increases, the $v_{c}^{\prime }$ is increased but the $v_{d}^{\prime }$ is decreased. However, even if the intersection angle increases, the $v_{c}^{\prime }$ does not always increase. The intersection angles have a decisive effect on the $v_{c}^{\prime }$ in a sine wave form. In other words, the $v_{c}^{\prime }$ increases when *θ* < 90°, but decreases when *θ* > 90°. Therefore, when *θ* is 60° and 120°, it has the same value. However, when the intersection angle (*θ*) of the continuous phase channel increases, the $v_{d}^{\prime }$ is decreased. This result can be described by Equation (10). The intersection angles have a decisive effect on the $v_{d}^{\prime }$ in a cosine wave form. Therefore, the intersection angle affects $v_{c}^{\prime }$, $v_{d}^{\prime }$, and droplet diameter. When the intersection angle is increased, the $v_{c}^{\prime }$ is increased, but the $v_{d}^{\prime }$ and droplet diameter are decreased.

**Figure 7:**
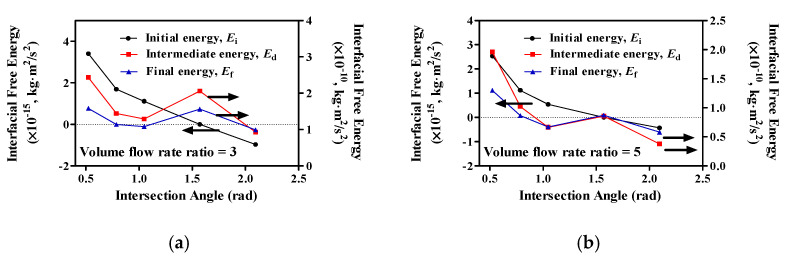
The relationship between intersection angle and interfacial free energy. (**a**) *v_c_/v_d_* = 3; (**b**) *v_c_/v_d_* = 5.

The droplet diameter is decreased when the intersection angle increases; however, when the intersection angle is 90°, the droplet diameter increases. These results cannot be explained by the modification of flow rate. Therefore, we tried to describe them using the interfacial free energy in the device (Figure 7).

We then determined the relationship between the intersection angle and interfacial free energy for volume flow rate ratios of 3 and 5 (Figure 7). A schematic view of a droplet generation device is shown in Figure 1. Two fluids are fed in from the left-hand side, one of which is to be dispersed. To the right, the dispersed phase is present as isolated droplets within the continuous phase. The task of the device is, thus, to decompose the singly connected liquid that is to be dispersed into a set of many unconnected regions of equal volume. It is clear from the start that the dispersion process is accompanied by an injection of considerable amounts of energy into the system, in order to account for the excess free energy of the interfaces separating the dispersed from the continuous phase in the emulsion that is ultimately produced. In Figure 1, *E_i_* corresponds to the initial energy state, *E*_f_ corresponds to the final energy state, and *E*_d_ corresponds to the droplet generation state (intermediate energy). These energies can be described using Equations (9)–(11). Our modeling results showed that *E_d_* has the highest energy level. *E_i_* decreased when the intersection angle increased, with *E_d_* and *E_f_* also decreasing. However, *E_d_* and *E_f_* showed decreases up to 60°, but showed increases at 90°, followed by decreases when *θ* > 90°.

We can determine the relationship between the droplet diameter and intersection angle by these results. When the intersection angle increases, *E_d_* and *E_f_* decreased, and the droplet diameter also decreased. However, *E_d_* and *E_f_* increased when *θ =* 90°. When the intersection angle was 90°, the droplet diameter increased, because *E_d_* and *E_f_* increased.

Droplet formation comprises three major steps. First of all, an interface must be created between the two immiscible liquids fed into the system. Initially, this is an interface between two singly connected regions. It must, therefore, be deformed considerably in order to reach the final state, where the dispersed-phase liquid fills many disconnected regions. Since this final state will be reached by decay from a deformed interface, there must be an intermediate state within the device, with more interfacial free energy than is found the final state, which spontaneously decays into the latter.

For the device to operate properly, it is important that the interface stays topologically intact while transitioning from its formation state to the intermediate state with energy *E_d_*. When the intermediate state is reached, the interface must become unstable with respect to the deformation mode, which finally leads, after spontaneous decay, to the desired dispersed state. Reaching the intermediate state is, thus, defined by the occurrence of an unstable mode of interface deformation or a saddle point on the interfacial free energy hypersurface in Hilbert space. It is of great importance, not only for purposes of classification, but also in terms of the operational stability of the droplet generation device, to discuss the properties of this saddle point in some detail, particularly with respect to the spectrum of its unstable modes [30].

In the present study, we observed a change in droplet diameter according to the angle of intersection of the input channels of the droplet generator with computational simulation; however, this model must be validated. Therefore, this model must be compared with a previously validated result (experimental or theoretical) in a future study.

## 4. Materials and Methods

A linear level-set method was applied to estimate the unknown parameters using normal equations. With this approach, we were able to reduce the measurement errors and the computational time compared with a nonlinear procedure. The two-phase flow, level-set CFD module in the COMSOL Multiphysics^®^ (Version 4.4) was used for the numerical simulations. The simulations were carried out in a three-dimensional (3D) domain, and the geometric dimensions used in the simulations are shown in Figure 2. We created a schematic illustration of the various droplet generation devices: the 30° input channel droplet device, the 45° input channel droplet device, the 60° input channel droplet device, the 90° input channel droplet device, and the 120° input channel droplet device. The numerical simulations were performed using the COMSOL Multiphysics^®^ to solve the incompressible Navier–Stokes equations for a single-phase flow in various FF geometries. The 3D mesh was generated as an unstructured mesh containing tetrahedral elements. The generalized minimum residual linear solver, along with the incomplete LU preconditioner, was applied to solve the partial differential equation (PDE) problems. As the geometric structure of this droplet device is an FF type, the equivalent diameter of the continuous phase inlet channel is 50 µm. We assumed that the volume flow rate ranged from 1 to 30 µL/h, and that silicone oil flowed through their channels. The width of the dispersed phase inlet channel was 25 µm (*w_d_*), and we assumed that this channel had a constant volume flow rate of 1 µL/h. We assumed that the thickness of the device was 15 µm (*h*). After performing grid dependence studies with different grid resolutions, a numerical grid with 19,521 elements was adopted. We assumed that the continuous-phase fluid, silicone oil (Ultragrad 19, Edwards, Sanborn, NY, USA), was injected from the side inlet channels, while the dispersed-phase fluid, an aqueous solution (water), was injected from the center channel inlet. The solutions were modeled as incompressible Newtonian fluids, and the fluid properties were taken directly from the data provided by Nie et al. [8]. The channel walls were specified as wetted wall conditions with a constant contact angle for all cases.

## 5. Conclusions

In this paper, we studied the effects of intersection angles on droplet diameter using numerical simulation modeling. Our modeling results showed that the droplet diameter decreases when the intersection angle increases. Increasing the intersection angle causes an increase in the velocity, which obstructs the dispersed phase fluid flow, thereby reducing the droplet diameter. This can be described as a correlation of interfacial free energy. When the intersection angle increases, the intermediate energy deceases, causing a decrease in droplet diameter. Through this study, the droplet diameter can be changed by changing the abutment angle.

This study conducted a study on the change in droplet diameter according to the change in the angle of the intersection of the two fluids in the flow of the dispersed and continuous fluids in the microdroplet generator. Before the experiment, the change in droplet diameter was predicted using a simulation. Therefore, a conclusion was drawn. However, further conclusions must be reserved until an actual validation experiment is conducted in the future.

## Data Availability

Data sharing not applicable.
